# Incidence and cost of treatment-emergent comorbid events in insured patients with chronic hepatitis C virus infection: a retrospective cohort study

**DOI:** 10.1186/1472-6963-14-429

**Published:** 2014-09-24

**Authors:** Sandhya Sapra, Eunice Chang, Michael S Broder, Gilbert L’Italien

**Affiliations:** Bristol-Myers Squibb, 1146 Lawrenceville Road, Lawrenceville, NJ USA; Partnership for Health Analytic Research, LLC, 280 S. Beverly Drive, Suite 404, Beverly Hills, CA USA; Yale University School of Medicine, 333 Cedar Street, New Haven, CT USA

**Keywords:** Insurance claims, Retrospective study, Pegylated interferon alpha, Ribavirin

## Abstract

**Background:**

Treatment-emergent comorbid events (TECs) are common In patients initiating treatment with pegylated interferon alpha (PEG-IFN-alfa) and ribavirin for chronic hepatitis C virus (HCV) infection. The purpose of this study was to estimate the incidence and incremental cost of these events.

**Methods:**

In a retrospective cohort analysis of healthcare claims, we studied patients with HCV who were newly treated with PEG-IFN-alfa/ribavirin between 2006 and 2008. TECs were defined by new medical/pharmacy claims for predefined conditions in the 12 months after treatment initiation. The net incremental cost of the TECs was the difference between baseline and follow-up costs for these comorbidities and their treatment, excluding PEG-IFN-alfa/ribavirin costs.

**Results:**

Of 3,795 newly treated patients, 1,269 (mean age 50.2, 36.2% female) met the selection criteria. New TECs were common, with 61.6% of patients having ≥1 event. Anemia was identified in 29.2% of patients, fatigue in 16.4%, depression in 11.5%, and neutropenia in 11.0%. The mean incremental cost for the predefined TEC in the postindex period was $6,377 ($2,782 for medical and $3,595 for pharmacy claims).

**Conclusions:**

In an insured US cohort with chronic HCV infection, TECs with PEG-IFN-alfa/ribavirin were common and increased costs by approximately $6,000 per treated patient. This estimate may be conservative because it excludes indirect costs. Costs might increase with new regimens that include a protease inhibitor because additional TECs may be expected. Better-tolerated therapies that reduce the financial burden on the healthcare system and improve patient experience are needed.

## Background

Chronic hepatitis C virus (HCV) infection, with a worldwide prevalence of 2%-3% [1], causes substantial loss of life and reduces quality of life in those who are infected [2]. Although the screening of blood products has reduced the incidence of HCV infection in developed countries, there is a long latency period before the disease becomes symptomatic and thus large numbers of new cases will continue to be identified over the coming years. Estimates suggest that about half of the 3.1 million US patients infected with HCV are unaware of their infection and only a small fraction have been treated [3–5].

Under optimal conditions, treatment of chronic HCV infection with pegylated interferon alpha (PEG-IFN-alfa) and ribavirin dual therapy produces sustained virologic response (SVR) of above 50% in patients with HCV infection [6, 7]. For patients with the most common HCV genotype (GT) in the US, GT-1, 48 weeks of PEG-IFN-alfa/ribavirin results in an SVR in 45%-50%, whereas in patients with GT-2 and GT-3, 24 weeks of PEG-IFN-alfa/ribavirin results in an SVR in 80%. Nearly all patients who achieve a SVR are cured of infection [8] and have a reduced risk of hepatocellular carcinoma and death [9]. However, treatment with PEG-IFN-alfa/ribavirin causes numerous adverse events including fatigue, flulike symptoms, gastrointestinal disturbances, psychological symptoms, and hematologic abnormalities [10]. These adverse events lead to decreased adherence, dose reduction, and early discontinuation. Patients who are able to maintain at least 80% adherence to their drug regimen have the highest likelihood of achieving a SVR, but treatment-emergent comorbid events (TECs) commonly limit adherence [10–12].

In the initial treatment of HCV infection, the addition of a protease inhibitor (telaprevir or boceprevir) to PEG-IFN-alfa/ribavirin (triple therapy) significantly improved SVR [13, 14]. However, adverse events were reported at higher rates among users of triple therapy than among users of PEG-IFN-alfa/ribavirin [13, 14]. Although a small subset of patients receiving triple therapy who have a rapid response can receive a shorter course of treatment, the 47%-77% premature discontinuation rates of PEG-IFN-alfa/ribavirin reported in clinical care settings [15, 16] would likely be similar with triple therapy.

Premature discontinuation of treatment and nonadherence may cause viral resistance and increase costs. Although combination therapies to treat HCV may cost between $23,000 and $78,000 per year (depending on GT, patient weight, and drug selected) [17], treatment with PEG-IFN-alfa/ribavirin has been shown to be cost effective when patients are cured [18]. In addition, nonadherent patients have higher HCV-related medical (i.e., excluding medication) costs than adherent patients [19]. However, despite their frequency and effect on treatment success, little is known about the cost associated with adverse events due to treatment with PEG-IFN-alfa/ribavirin itself [20]. The objective of this study was to estimate the incidence of TECs and the incremental costs of treating these events in insured patients initiating PEG-IFN-alfa/ribavirin treatment for chronic HCV infection. Secondary objectives were to explore, in a managed care population, the rate of discontinuation of PEG-IFN-alfa/ribavirin therapy and the temporal pattern of the costs of these TECs.

## Methods

### Study design and data source

This study was a retrospective cohort analysis that used data from the i3 Ingenix LabRx database spanning the 4-year period from 7/1/05 to 6/30/09. This database is a Health Insurance Portability and Accountability Act (HIPAA)-compliant administrative claims database of 8–10 million covered lives, representing all major regions of the US. The database contains deidentified adjudicated pharmacy and medical claims submitted for payment by providers, healthcare facilities, and pharmacies and includes information on physician visits, medical procedures, hospitalizations, drugs dispensed, and tests performed. Healthcare charges are reported (medical, inpatient, and pharmacy) in this database, but paid claims and costs are not. Also available are member enrollment and benefit information as well as limited patient, provider, and hospital demographic information. Since the study did not involve direct contact with human subjects, did not involve an intervention, and did not involve collection of any identifiable patient information, Institutional Review Board (IRB) approval was not required.

### Study population

The study included treatment-naive patients with HCV infection who began treatment with PEG-IFN-alfa/ribavirin during a 2-year period between 7/1/06 and 06/30/08. PEG-IFN-alfa and ribavirin were identified using National Drug Codes. The date of the first medication fill for PEG-IFN-alfa/ribavirin within this period was defined as the index date. The 24-month study period included 12 months before and 12 months after the index date. Treatment-naive HCV-infected patients were defined as those who did not fill any prescriptions for PEG-IFN-alfa prescription for at least 12 months before the first such fill. HCV infection was defined by the presence of at least 1 medical claim with an ICD-9-CM (International Classification of Diseases, 9th Revision, Clinical Modification) code for HCV (070.41x, 070.44, 070.51, 070.54, 070.7x) during the preindex period.

Patients were excluded if they were <18 years of age or if they were not continuously enrolled during the 24-month study period. We also excluded individuals who initiated therapy at a dose not recommended by the manufacturer [21–24]. Finally, we excluded patients with certain medical claims during the preindex period, including those with an ICD-9-CM code for hepatitis B (070.2x, 070.3x),or hematologic malignancies for which interferon may have been indicated [leukemia (204.xx-208.xx), Hodgkin’s lymphoma (201.xx), non-Hodgkin’s lymphoma (200, 202.0-202.2, 202.8), multiple myeloma (203.0-203.1, 238.6), acute lymphocytic leukemia (204.0), chronic lymphocytic leukemia (204.1), acute nonlymphocytic leukemia including acute myeloid leukemia (205.0), acute monocytic leukemia (206.0), chronic myeloid leukemia (205.1), or other leukemias (204.2, 204.8-204.9, 205.2, 205.8-205.9, 206.1-206.2, 206.8-206.9, 207.8, 208.0-208.2, 208.8-208.9)].

### Outcome measures

The primary outcome variable was net incremental cost, which was calculated as the difference between preindex and postindex cost for a prespecified list of TECs (see following paragraph) and their treatments, excluding the cost of PEG-IFN-alfa/ribavirin therapy. Charges for TECs were taken from medical claims consistent with one of these events or pharmacy claims with National Drug Codes for medications to treat the events. Charges for visits lacking a code for one of the listed TECs were not included. New TECs were defined by a medical or pharmacy claim in the postindex period that was not present in the preindex period.

TECs were grouped as blood disorders (anemia, neutropenia, thrombocytopenia), gastrointestinal disorders (nausea/vomiting, diarrhea), endocrine disorders (diabetes, hyperthyroidism, hypothyroidism), psychiatric disorders (depression, anxiety disorders, bipolar disorders, insomnia), skin and subcutaneous disorders (alopecia, skin rash), and other disorders (dyspnea, fatigue, headache). Medications used to treat TECs were grouped similarly and included those to treat blood disorders (epoetin alfa, darbepoetin, filgastrim, and eltrombopag), endocrine disorders (antidiabetes medications and thyroid agents), psychiatric disorders (anxiolytics, antidepressants, antipsychotics/antimanics, and hypnotics), skin disorders (topical steroids), and other (antimigraine).

Secondary outcomes included the incidence of new TECs, timing of such TECs, and the rate of discontinuation of PEG-IFN-alfa/ribavirin therapy. PEG-IFN-alfa/ribavirin therapy was considered to be discontinued if there were no prescription fills for both medications for at least 60 days. The date of discontinuation was defined as the last day the patient had both PEG-IFN-alfa and ribavirin available, based on the days of supply as reported in the pharmacy claims. If PEG-IFN-alfa and ribavirin were discontinued on different dates, the earlier date was used.

### Covariates

Other measures included patient age, gender, race, geographic region, and the presence of human immunodeficiency virus (HIV) infection/acquired immune deficiency syndrome (AIDS). Because physician specialty may impact cost and resource use, we identified the specialty of each patient’s usual care physician using a validated method. The specialty of the physician prescribing therapy was not directly identifiable in the claims database. Instead, the method counts all claims for evaluation and management services and identifies the physician specialty with the largest plurality of such claims [25]. Physician specialty was assigned using claims from the preindex period.

### Sensitivity analyses

The recommended PEG-IFN-alfa/ribavirin treatment duration is either 24 or 48 weeks, depending on HCV GT. Claims data are used to process payments and generally do not contain clinical information such as test results. However, GT data were available for a small subset of study subjects and we thus explored discontinuation rates for this subset in a sensitivity analysis. In another sensitivity analysis, we restricted the group of patients analyzed to those who were treated beyond 28 weeks of PEG-IFN-alfa/ribavirin treatment, under the assumption that they had GT-1 infection.

### Statistical analysis

For descriptive analysis, percentages, medians, means, and standard deviations were calculated for all baseline variables. We reported the percentage of patients with any new TEC as well as the percentage with a new event for each TEC. We reported the mean and standard deviations for TEC charges in both the pre- and postindex periods and for net incremental TEC charges. Treatment discontinuation was reported as the proportion of patients who no longer had PEG-IFN-alfa/ribavirin available at successive 4-week intervals. Charges for TECs were also stratified by duration of therapy. All data transformations and statistical analyses were performed using SAS version 9.2 (SAS Institute, Cary, NC).

## Results

We identified 3,795 patients with HCV infection who were newly treated with PEG-IFN-alfa/ribavirin during the study period. Of these, 1,269 met the inclusion criteria (Table 1), The most common reason for exclusion (2,274 patients) was lack of continuous enrollment for 24 months. One hundred thirty-eight patients were excluded because of a co-occurring diagnosis of hepatitis B or a hematologic malignancy, 16 because they lacked an HCV diagnosis in the pre-index period, and 85 because they had an initial dose of PEG-IFN-alfa/ribavirin other than one of those recommended by the manufacturer. Most of the included subjects were male (63.8%), with 85.1% between the ages of 40 and 59 years old (mean age, 50.2 years). Over half (55.6%) the patients were from the South. Only 3.5% of patients in this sample were coinfected with HIV. The most common treating physician specialty was gastroenterology (32.4%), followed by family practice (28.5%) and internal medicine (25.2%).

**Table 1:** **Characteristics of insured patients initiating PEG-IFN-alfa and ribavirin treatment for chronic HCV infection**

Characteristic	No. (%) of insured patients (n = 1269)
**Age, mean (SD), y**	50.2 (7.7)
**Age group (y)**	
18-29	31 (2.4)
30-39	61 (4.8)
40-49	405 (31.9)
50-59	675 (53.2)
60-69	91 (7.2)
70+	6 (0.5)
**Female**	459 (36.2)
**Region**	
East	123 (9.7)
Midwest	242 (19.1)
South	705 (55.6)
West	199 (15.7)
**Type of health benefits**	
Commercial	1,244 (98.0)
Medicaid-HMO	25 (2.0)
**HIV/AIDS**	44 (3.5)

AIDS, acquired immune deficiency syndrome; HCV, hepatitis C virus; HIV, human immunodeficiency virus; PEG-IFN-alfa, pegylated interferon alpha.

**Figure 1:**
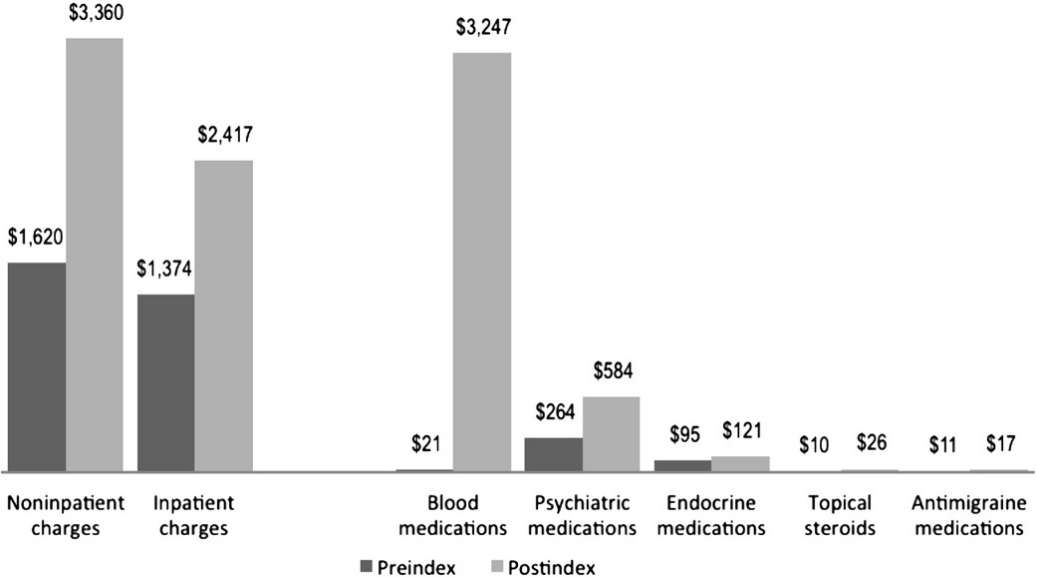
**Increase in non-medication-related and medication-related charges for treatment-emergent comorbid events between preindex and postindex periods.**

**Figure 2:**
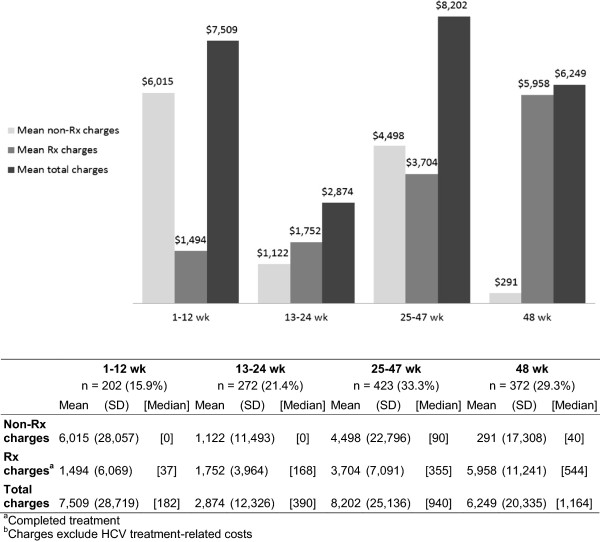
**Increase in charges from 1-year preindex to 1-year postindex periods for treatment-related comorbid events, by treatment duration.** Data on four mutually exclusive groups are presented: those who discontinued PEG-IFN-alfa/ribavirin between weeks 1–12, weeks 13–24, weeks 25–47, and after week 47. For all groups, annual charges are shown.

Table 2 shows the proportion of patients who had a diagnosis recorded for each TEC before beginning treatment (preindex period) and the proportion with a diagnosis recorded for the TEC while they were receiving treatment (postindex period). In addition, the proportion of patients who had a new diagnosis while receiving treatment (e.g., no such diagnosis in the preindex period, followed by a diagnosis in the postindex period) is shown. These newly diagnosed conditions are considered to be TECs. Disorders of blood were observed in 14.3% of patients in the preindex period and in 41.2% of patients in the postindex period. In the postindex period, 35.2% of patients had a new diagnosis of a blood disorder and new psychiatric events were observed in 20.1%, endocrine events in 12.1%, and gastrointestinal events in 12.0%. Most (61.6%) patients had at least 1 newly diagnosed TEC. The most common TECs were anemia (29.2%), fatigue (16.4%), depression (11.5%), neutropenia (11.0%), insomnia (8.98%), nausea/vomiting (8.35%), skin rash (7.64%), hypothyroidism (6.93%), thrombocytopenia (6.70%), dyspnea (5.99%), and headache (5.91%).The mean net incremental charge for the predefined TEC in the postindex period was $6,377, comprising $2,782 for medical and $3,595 for pharmacy claims. The largest component of the incremental increase in pharmacy costs was for medications to treat anemia and neutropenia (mean increase, $3226; Figure 1). These medications included epoetin alfa, darbepoetin, filgastrim, and eltrombopag. The increase in non-drug-related charges was greatest in patients who completed only 12 weeks of PEG-IFN-alfa/ribavirin treatment (mean: $6,015; range: -28,831 to 291,838) and least in patients who completed the 48-week treatment (mean: $291; range: -208,060 to 95,782).Two hundred two patients (15.9%) discontinued PEG-IFN-alfa/ribavirin treatment by 12 weeks. The mean incremental TEC charge for this group was $7,509 (range: -29,273 to 292,904). The mean incremental charge was $8,202 (range: -57,384 to 226,864) in the 423 patients who continued treatment for between 25 and 47 weeks and $6,249 (range: -207,548 to 130,297) in the 372 patients who completed 48 weeks of PEG-IFN-alfa/ribavirin treatment (Figure 2).

**Table 2:** **Frequency of various treatment-emergent comorbid events** ^**a**^ **in insured patients initiating PEG-IFN-alfa and ribavirin treatment for chronic HCV infection**

Variable	No. (%) with treatment-emergent comorbid event in the preindex period	No. (%) with treatment-emergent comorbid event in the postindex period	No. (%) diagnosed with a new treatment-emergent comorbid event in the postindex period ^b^
	(n = 1269)	(n = 1269)	(n = 1269)
**Any treatment-related comorbid event**	803 (63.3)	923 (72.7)	782 (61.6)
**Blood**	182 (14.3)	523 (41.2)	446 (35.2)
Anemia	147 (11.6)	461 (36.3)	371 (29.2)
Neutropenia	18 (1.42)	144 (11.4)	139 (11.0)
Thrombocytopenia	46 (3.62)	101 (7.96)	85 (6.70)
**Gastrointestinal**	114 (8.98)	175 (13.8)	152 (12.0)
Nausea/vomiting	78 (6.15)	124 (9.77)	106 (8.35)
Diarrhea	55 (4.33)	75 (5.91)	63 (4.96)
**Endocrine**	317 (25.0)	354 (27.9)	154 (12.1)
Diabetes	214 (16.9)	217 (17.1)	61 (4.81)
Hyperthyroidism	20 (1.58)	30 (2.36)	26 (2.05)
Hypothyroidism	117 (9.22)	164 (12.9)	88 (6.93)
**Psychiatric**	262 (20.7)	375 (29.6)	255 (20.1)
Depression	181 (14.3)	266 (21.0)	146 (11.5)
Anxiety disorders	48 (3.78)	54 (4.26)	31 (2.44)
Bipolar disorders	22 (1.73)	27 (2.13)	17 (1.34)
Insomnia	82 (6.46)	143 (11.3)	114 (8.98)
**Skin and subcutaneous**	59 (4.65)	119 (9.38)	111 (8.75)
Alopecia	11 (0.87)	17 (1.34)	15 (1.18)
Skin rash	49 (3.86)	103 (8.12)	97 (7.64)
**Other disorders**	383 (30.2)	423 (33.3)	311 (24.5)
Dyspnea	91 (7.17)	98 (7.72)	76 (5.99)
Fatigue	270 (21.3)	319 (25.1)	208 (16.4)
Headache	96 (7.57)	100 (7.88)	75 (5.91)

HCV, hepatitis C virus; PEG-IFN-alfa, pegylated interferon alpha.

^a^ICD-9-CM diagnosis code for listed events appearing in any diagnosis field.

^b^Event appears during period when patient is being treated but not in pretreatment period.

Considering all study subjects, 14.2% discontinued treatment before week 12, 32.8% before week 24, and 70.7% before week 48 (Table 3). In a sensitivity analysis restricted to 238 patients for whom HCV GT was known, 81.1% (n = 193) had GT-1/4/6 and therefore these patients should have received 48 weeks of PEG-IFN-alfa/ribavirin. In this group of 193 patients, 14.5% discontinued treatment before week 12, 31.6% before week 24, and 62.7% before week 48. In a second sensitivity analysis restricted to patients treated beyond 28 weeks, and therefore presumed to have predominately GT-1 infection, 39.8% discontinued PEG-IFN-alfa/ribavirin treatment before week 48.

**Table 3:** **Duration and discontinuation of PEG-IFN-alfa/ribavirin therapy in insured patients receiving treatment for chronic HCV infection**

Patient group		Duration of PEG-IFN-alfa/ribavirin therapy (days)			Discontinue before 12 wk		Discontinue before 24 wk		Discontinue before 48 wk	
	No. of patients	Mean	(SD)	Median	n	(%)	n	(%)	n	(%)
All	1,269	214.8	(113.8)	195	180	(14.2)	416	(32.8)	897	(70.7)
Patients with known GT	238	223.1	(115.3)	211	32	(13.4)	79	(33.2)	165	(69.3)
GT 2/3	45	161.3	(60.4)	168	4	(8.9)	18	(40.0)	44	(97.8)
GT 1/4/6	193	237.5	(120.2)	282	28	(14.5)	61	(31.6)	121	(62.7)
Patients treated >28 weeks^a^	618	315.6	(57.1)	339	–	–	–	–	246	(39.8)

GT, genotype; HCV, hepatitis C virus; PEG-IFN-alfa, pegylated interferon alpha.

^a^Presumed to have predominately GT-1 infection.

## Discussion

In an insured US cohort with chronic HCV infection who were treated with PEG-IFN-alfa/ribavirin, most patients experienced one or more TEC. The most commonly identified TECs were anemia, fatigue, depression, and neutropenia. Anemia was identified in 29.2% of patients in this study, compared with in 22% of patients in clinical trials. Rates of several adverse events including fatigue (16%), insomnia (9%), and depression (12%) were lower than those observed in clinical trials (54%, 37%, 22%, respectively) [10]. This may be expected since patients in clinical practice are typically monitored less aggressively than those in clinical trials. We used ICD-9-CM codes to identify TECs, and thus we only identified events that warranted an interaction with the healthcare system in the form of an office visit, hospitalization, or prescription. In addition, even when identified clinically, ICD-9-CM codes may not be recorded for less-severe events. As a result, the frequency and cost of TECs we report may be underestimated.

Despite this likely downward bias, we estimated that TECs increased direct treatment costs by 25% ($6,377), with just over half of charges from prescription medications and the rest from office visits, hospitalizations, and other nonprescription charges. Although we did not assess indirect costs in this study, several of the TECs (e.g., anemia, depression, and insomnia) experienced by patients who receive PEG-IFN-alfa/ribavirin therapy have associated indirect costs. For example, in studies of patients with anemia due to chronic kidney disease, hemoglobin values were positively correlated with days worked [26, 27]. In a study that reviewed healthcare claims from employees at a major US corporation, nearly 20% of the costs of depressive illness were related to disability, and this study did not take into account lost productivity or sick leave [28]. Depressed individuals took significantly more time off work than those with diabetes, heart disease, or back problems [28]. In addition, absenteeism and disability expenditures were higher in individuals with insomnia than those without it [29]. Together these studies suggest that indirect costs of these TECs are likely to be significant.

Shortened treatment duration was not associated with a reduced cost of treating TECs. Nonprescription costs accounted for most of the total costs in patients who stopped treatment by 12 weeks and between 24 and 48 weeks, suggesting that these groups of patients frequently experience TECs. The lowest cost of treating TECs was for patients who stopped treatment between 13 and 24 weeks; most of these patients probably discontinued treatment because they completed treatment for GT 2/3 infection rather than because of TECs. Patients who completed therapy at 48 weeks had the lowest nonprescription costs but high prescription costs, possibly indicating stable management of TECs such as anemia or neutropenia with costly growth factors.

Although comparisons to other studies are difficult because of differences in methodology, the mean treatment duration of 215 days is consistent with estimates from other studies of 172–240 days [15, 16, 19, 30]. In a retrospective cohort study of Veterans Affairs patients, two of the most common TECs we identified, anemia and neutropenia, were associated with lower persistence with PEG-IFN-alfa/ribavirin treatment (mean 172 days) [30]. Similar to patients in our study, the investigators identified patients with anemia and neutropenia through both ICD-9-CM codes and by identifying medication use (specifically the use of growth factors). Growth factors may improve treatment tolerability and thus enhance persistence. Dissimilarities in data sources, clinical care settings, data analysis techniques, and patient characteristics may explain some of the differences in the treatment discontinuation estimates. Although we could not distinguish between patients who discontinued therapy because of adverse events and those who discontinued therapy because of lack of virologic response, our findings support the concept that adverse events lead to high rates of therapy discontinuation.

Limitations of this study include those common to claims analysis. A commercially insured population may not be representative of the entire US population nor of treatment patterns in other countries. Lack of clinical data can confound interpretation. In particular, HCV treatment duration is dictated by GT, which was unavailable for most patients in this study. HCV GT was available for a subset of 238 patients. In this subset, 81% had GT 1/4/6, which is similar to other US cohorts, suggesting that our data are representative of the US population [31, 32]. According to standard treatment recommendations, these patients with predominately GT-1 would be expected to complete 48 weeks of treatment [33]. This subset of patients had discontinuation rates that were similar to the overall group, which suggests that our assumption that treatment duration was indicative of GT was reasonable. Furthermore, response guided therapy would lead some patients to have treatment recommended beyond 48 weeks. If these patients continued treatment up to 48 weeks, they might be clinically discontinuing therapy prematurely, whereas in our analysis they would not be considered to have done so. Conversely, discontinuation of therapy may be recommended when virologic response is poor, and we could not distinguish between this and TECs as a cause of discontinuation*.* This study was intended to examine only one aspect of cost, not total treatment costs or cost effectiveness. HCV treatment with PEG-IFN-alfa/ribavirin costs between $23,000 and $78,000 per year, and newer treatments are substantially more expensive [17]. Given the timing of this study, none of the patients were treated with newer direct acting antivirals.

In an additional sensitivity analysis of the patients who stopped therapy after 28 weeks and were thus presumed to have GT 1/4/6, 40% stopped before completing the recommended duration of therapy. Patients treated over 12 weeks with GT-1 would be expected to have had a virologic response [33] and thus discontinuations after 24 weeks would most likely be due to adverse events in those with GT-1. Overall, these sensitivity analyses further support the idea that discontinuation rates in patients treated with PEG-IFN-alfa/ribavirin are high and frequently due to TECs.

Additional limitations include miscoding or undercoding of claims, which may affect the accuracy of cost estimates. Finally, although we were only able to study patients who were receiving double therapy, the cost of TECs may rise with the use of triple therapy (PEG-IFN-alfa/ribavirin plus a protease inhibitor) because gastrointestinal events, skin rash, and anemia are more common with triple therapy than with PEG-IFN-alfa/ribavirin alone [10, 14, 34].

## Conclusion

Treatment of chronic HCV infection with PEG-IFN-alfa/ribavirin led to frequent TECs and that these events were associated with significant costs. Furthermore, nearly half of treated patients discontinued therapy. It is likely that adding a protease inhibitor to the PEG-IFN-alfa/ribavirin treatment discussed in this study will result in similar or increased TECs. Thus, better-tolerated therapies associated with fewer TECs that reduce healthcare system costs and improve patient continuation rates are needed.
